# Anti-Atherosclerotic Effect of Afrocyclamin A against Vascular Smooth Muscle Cells Is Mediated via p38 MAPK Signaling Pathway

**DOI:** 10.22074/cellj.2021.7148

**Published:** 2021-05-26

**Authors:** Yan Gu, Zhanzhan Xiao, Jianlie Wu, Mingjin Guo, Ping Lv, Ning Dou

**Affiliations:** 1 Department of Vascular Surgery, Tianjin First Center Hospital, Tianjin, China; 2 Department of Emergency Services, The Fourth People’s Hospital of Jinan City, Jinan, Shandong Province, China; 3 Department of Vascular Surgery, The Affiliated Hospital of Qingdao University, Qingdao City, China; 4 Department of Hematology, The Fourth People’s Hospital of Jinan City, Jinan, Shandong Province, China; 5 Department of General Surgery, Shanghai Fourth People's Hospital Affiliated to Tongji University School of Medicine, Shanghai, China

**Keywords:** Afrocyclamin A, Cardioprotective, Pro-Inflammatory Cytokines, p38 Mitogen-Activated Protein Kinase

## Abstract

**Objective:**

Research suggests that fine particulate matter (PM2.5) contributes to the expansion and development of atherosclerosis. Infiltration and proliferation of vascular smooth muscle cells (VSMCs) from the blood vessel media into the intima, is an important step in the atherosclerosis pathophysiology. Afrocyclamin A, is an oleanane-type triterpene saponin, isolated from *Androsace umbellate*, which is commonly used in Chinese herbal medicine. In the study, we examined the effect of Afrocyclamin A on PM2.5-induced VSMCs proliferation and scrutinized possible mechanisms of action.

**Materials and Methods:**

In the experimental study, counting Kit-8 (CCK-8) assay was used for estimation of VSMCs viability. BrdU immunofluorescence was used for estimation of VSMCs proliferation. The levels of antioxidant parameters such as malonaldehyde (MDA), superoxide dismutase (SOD), and glutathione (GSH); proinflammatory cytokines such as interleukin-1β (IL-1β), IL-6, tumor necrosis factor-α (TNF-α), nitric oxide (NO), endothelin-1 (ET-1), and vascular cell adhesion molecule-1 (VCAM-1), were estimated. The expression of proliferating cell nuclear antigen (PCNA) and phospho-p38 MAPK (p-p38 MAPK) was assessed.

**Results:**

Compared to PM2.5-treated cells, in addition to reducing PM2.5-induced VSMCs proliferation, Afrocyclamin A reduced the expression of PCNA and p-p38 MAPK, down-regulated the level of TNF-α, IL-1β, IL-6, VCAM-1, MDA and ET-1, and up-regulated SOD, GSH and NO level. Furthermore, the anti-proliferative effect of Afrocyclamin A was considerably increased following co-incubation of Afrocyclamin A with SB203580 (p38 MAPK inhibitor) in comparison with Afrocyclamin A-treated cells.

**Conclusion:**

Based on the results, we can conclude that Afrocyclamin A might reduce PM2.5-induced VSMCs proliferation via reduction of p38 MAPK signaling pathway.

## Introduction

Previous researches suggested that the particulate matter less than 2.5 μm (PM2.5) air pollution exposure is related with overall cardiovascular mortality, cardiovascular disease (CVD) and mortality and long-term exposure of PM2.5 was found to be related to the risk of atherosclerosis, the underlying pathology of CVD (1). According to the American Heart Association, PM2.5 accelerates the expansion of atherosclerosis and ischemic disease (2). It was exhibited that exposure to PM2.5 for 1 year is positively concomitant with the carotid intima-media thickness in the general population, which is considered a significant index of subclinical atherosclerosis and contributes to the expansion of the atherosclerotic vascular disease (3). Furthermore, an *in vivo* study demonstrated that PM2.5 could induce systemic inflammation and oxidative stress and contributes to the expansion of atherosclerosis. Nevertheless, the underlying mechanisms of PM2.5-induced atherogenesis has not been fully explained.

Vascular smooth muscle cells (VSMCs) are considered the key constituent of the blood vessel wall and essential regulators of vascular function (4). Physiologically, VSMCs also help to regulate the blood flow, maintain the vascular tone, circulate the oxygen and equally distribute the nutrients. Moreover, during the arterial restenosis and atherogenesis, the biology of VSMCs is altered. VSMCs modify the contractile phenotype to the proliferate abnormally, synthetic phenotype and synthesize extracellular matrix proteins, which play a crucial role in the intimal hyperplasia and development of vascular injury

Studies suggested that atherosclerosis pathogenesis and neo-intimal thickening post-angioplasty involve excessive proliferation and migration of smooth muscle cells (SMCs) from media into the blood vessels (4, 5). It is well documented that enhanced expressions of various factors such as platelet-derived growth factor (PDGF) and basic fibroblast growth factor (bFGF) also take part in the formation of atheroma (6). The above discuss agonists activate the mitogen-activated protein kinase (MAPK) and phosphoinositide 3-kinases (PI-3) pathway and uphold proliferation and migration of VSMCs leading to their consequent deposition in the plaque. In the current experimental investigation, we scrutinized the anti-atherosclerotic effect of Afrocyclamin A on PM2.5- induced VSMCs proliferation and explored the underlying mechanism.

## Materials and Methods

 Afrocyclamin A was received as a gift sample. Bafilomycin A1, 3-Methyladenine (3-MA), ammonium chloride and chloroquine were purchased from the Sigma Aldrich, USA. Transforming growth factor beta 1 (TGF-β1) as purchased from Peprotech Inc. (Rocky Hill, NJ, USA). Pro-inflammatory cytokines such as tumor necrosis factor-α (TNF-α), interleukin-1β (IL-1β), IL-6 were purchased from the eBioscience (San Diego, CA, USA). Counting Kit-8 (CCK-8) (CK04) was purchased from Dojindo Molecular Technologies, Inc., USA; superoxide dismutase (SOD), glutathione (GSH), malonaldehyde (MDA), catalase (CAT) and nitric oxide (NO) were purchased from Jiancheng Bioengineering Institute (Nanjing, China). Collagen, type, α-actin, microtubule-associated protein 1light chain 3 (LC3), β-catenin and histone antibodies were purchased from Santa Cruz Biotechnology (Santa Cruz, CA). Antibodies for Beclin-1, Atg5 and osteocalcin were purchased from Epitomics (Burlingame, CA, USA).

### *In vitro* study

### Collection and preparation of PM2.5

PM2.5 samples were prepared based on a previously reported method with minor modifications (4). Briefly, Zefluor PTFE membrane filters were used for the collection of PM2.5 samples using the low volume particle samplers. PM2.5 samples were extracted from the filters by soaking for 30 minutes in ultra milli-Q water followed by sonication for 60 minutes. After that, arotary evaporator was used to concentrate the extracts which were then filtered through a Teflon membrane and kept in a dark place at -20ºC to maintain the chemical stability until assayed. 

### Cell culture

Human aortic VSMCs were purchased from Chinese Academy of Science Cell Bank (Shanghai, China). Dulbecco’s Modified Eagle Medium (DMEM) supplemented with fetal bovine serum (FBS, 10%) and antibiotics (100 μg/ml streptomycin and 100 μg/ml of penicillin) was used for the culture of the VSMCs. In order to scrutinize VSMCs proliferation induced by PM2.5, the cells were treated with different concentration of Afrocyclamin A for 24 hours (7). To further scrutinize the effect and potential mechanism of Afrocyclamin A on PM2.5-induced VSMCs proliferation, cells were treated with different concentrations of Afrocyclamin A of p38 MAPK inhibitor for 1 hour and followed by the addition of PM2.5 for 24 hours. 

### Estimation of cell viability

The cells were seeded at a density of 1×10^4^ /well in 96- well plates and cultured at 37˚C in CO_2_ (5%) incubator for 24 hours. After that, the medium was successfully replaced with the serum-free medium for the next 24 hours. After the above-discussed treatment, the medium was again replaced with the medium containing CCK-8 (10 μl) for 2 hours. Another one blank wells were performed with containing the CCK-8 (10 μL). Finally, the absorbance was read at 540 nm using nanodrop reader. Cell proliferation was estimated according to the following formula: 

Cell viability=[A (PM2.5)−A (blank)]/[A (PBS)−A (blank)].

### Biochemical and antioxidant parameters

The levels of MDA and SOD were estimated using colorimetric assay kits. The level of NO was estimated in the VSMCs culture supernatant using the nitrate reductase method according to the manufacturer’s instructions. Radioimmunoassay technique was used for the estimation of ET-1 based on the manufacturer’s instruction.

### *In vivo* study

### Animal

A total 30 Wistar rats (100-150 g) were used for the experimental study. The rats were received from the institute animal house. The rats were kept in the polyethylene cages under standard conditions (temperature 22 ± 3ºC; 60 ± 5 relative humidity) and they received standard diet and water ad libitum. The rats were acclimatized 7 days before the experimental study. The current experimental study was approved by the institutional animal Ethical Committee (202008-1006).

### Cell culture and treatment

Wistar rats were sacrificed, the aorta was successfully removed and the VSMCs were isolated as previously reported (8, 9). The isolated VSMCs were cultured in the DMEM supplemented with FBS (10%) and maintained under CO_2_ (5%) at 37˚C in a humidified atmosphere. The cells were cultured in the DMEM and the expression of known marker protein α-actin was assessed using an immunofluorescence assay. After that, the VSMCs were washed with phosphate buffered saline (PBS) and re-cultured in the serum-free medium for the next 24 hours, before stimulation by TGF-β. Various concentrations of afrocylamin A were used for further experiments. 

### Transfection of vascular smooth muscle cells

For the over-expressed the expression of β-catenin in VSMCs, cells were transferred with either empty vector or the same vector containing a cDNA encoding wild type β-catenin. Briefly, the cells were cultured in the plates and grown for 24 hours until they reached 50-60% confluence. Then VSMCs were transfected with WT β-catenin or empty vector using the transfection reagent based on the manufacturer’s instructions. 

### Cell viability assay

MTT assay was used to assess cell viability. Here, 5×10^3^ cells were seeded in the 96-well plates overnight. After that, the cells were treated with the test drug and incubated with 3-(4,5-dimethylthiazol-2-yl)-2,5-diphenyl tetrazolium bromide (MTT, 5 mg/ml) for 3 hours and subsequently, solubilized in dimethyl sulfoxide (DMSO, 200 μl). Finally, the absorbance was read at 570 nm using an enzyme-linked immunosorbent assay (ELISA) reader. 

### Calcification analysis

To estimate cell calcification, QuantiChromTM Calcium Assay Kit (Bioassay Systems, Hayward, CA) was used for the estimation of calcium content. The absorbance was read at 612 nm using an ELISA reader. 

### Nuclear and cytosolic fractionation

After culturing VSMCs, the cells were washed with ice-cold PBS. A previously reported method was used for the extraction of cytosolic and nuclear protein with minor modifications. Briefly, VSMCs were harvested in the hypotonic lysis buffer and incubated on the ice for 5 minutes. After that, the cell lysate was chilled for 10 minutes on ice and then, vigorously shaken in the presence of Nonidet P-40 for 10 minutes and centrifuged for separating the nuclear fraction. The supernatants containing the cytosolic protein were collected. For the collection of nuclear fractionation, the high salt buffer was added to the extract with continuous shaking and the extract was centrifuged for collection of the supernatants. 

### Statistical analysis

Data was analyzed by ANOVA, followed by Tukey’s post hoc test, using the Graphpad Prism 7 version software (USA). Data is presented as means ± SEM. A value of P<0.05 was considered significant.

## Results

### Effect of Afrocyclamin A on PM2.5-induced proliferation in vascular smooth muscle cells

**Fig.1:**
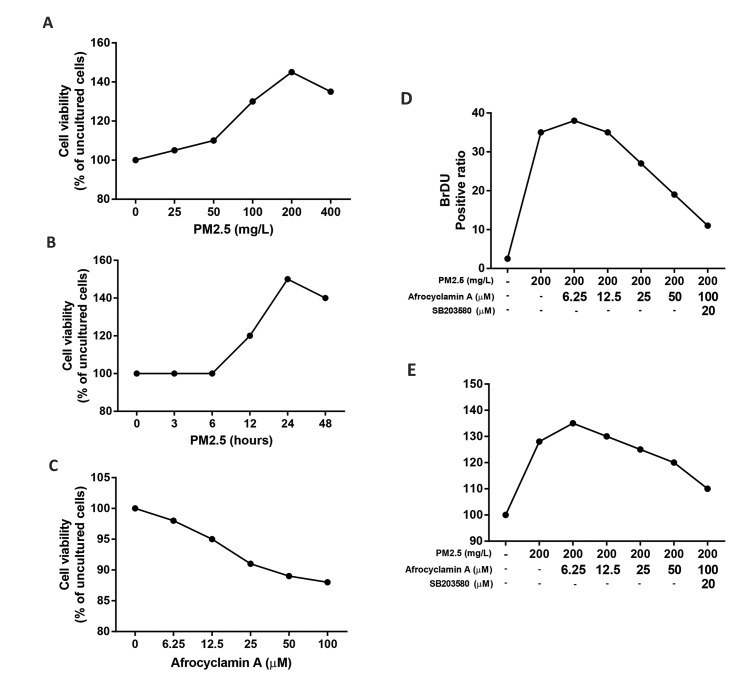
Effect of Afrocyclamin A on VSMCs proliferation. **A.** Cell were increased after 24 hours. **B.** Cells were stimulated with PM2.5 (200 mg/l) at different time intervals, **C. **Cells were treated with different concentrations of Afrocyclamin A (0, 6.25, 12.5, 25, 50 and 100 µM), **D. **Cells were treated with different concentrations of Afrocyclamin A and SB203580 (p38MAPK inhibitor) for 1 hour, followed by addition of PM2.5 (200 mg/l) for 24 hours, and **E. **Cells were treated with Afrocyclamin A and SB203580 (p38 MAPK inhibitor) for 1 hour, followed by addition of PM2.5 (200 mg/l). VSMCs; Vascular smooth muscle cells.

BrdU immunofluorescence and CCK-8 assay kits were used for estimation of cell proliferation. Figure 1A shows that the PM2.5 (200 mg/l) increased VSMCs cell viability. Figure 1B shows that PM2.5 (200 mg/l) exposure for 24 hours led to a considerable enhancement of VSMCs viability. Figure 1C demonstrates that Afrocyclamin A treatment (0~50 μM) for 24 hours compared to the untreated cells. Moreover, PM2.5 (200 mg/l)-treated cells pretreated with Afrocyclamin A (50 μM) were considered in subsequent experiments. PM2.5 considerably enhanced VSMCs viability as compared to the untreated cells, which was inverted by Afrocyclamin A in a dose-dependent manner. The anti-proliferative effect of Afrocyclamin A was increased via SB203580 as compared to Afrocyclamin A-treated cells (Fig .1D). The results showed that the pro-proliferative effect of PM2.5 on VSMCs, was reversed by Afrocyclamin A treatment. As shown by the BrdU immunofluorescence assay, PM2.5 (200 mg/l) considerably enhanced VSMCs proliferation as compared to the untreated cells (Fig .1E). Afrocyclamin A reduced PM2.5-induced proliferation of VSMCs, and the antiproliferative potential of Afrocyclamin A was increased by SB203580 administration as compared to Afrocyclamin A-treated cells. 

#### Effect of Afrocyclamin A on antioxidant parameters on vascular smooth muscle cells

**Fig.2:**
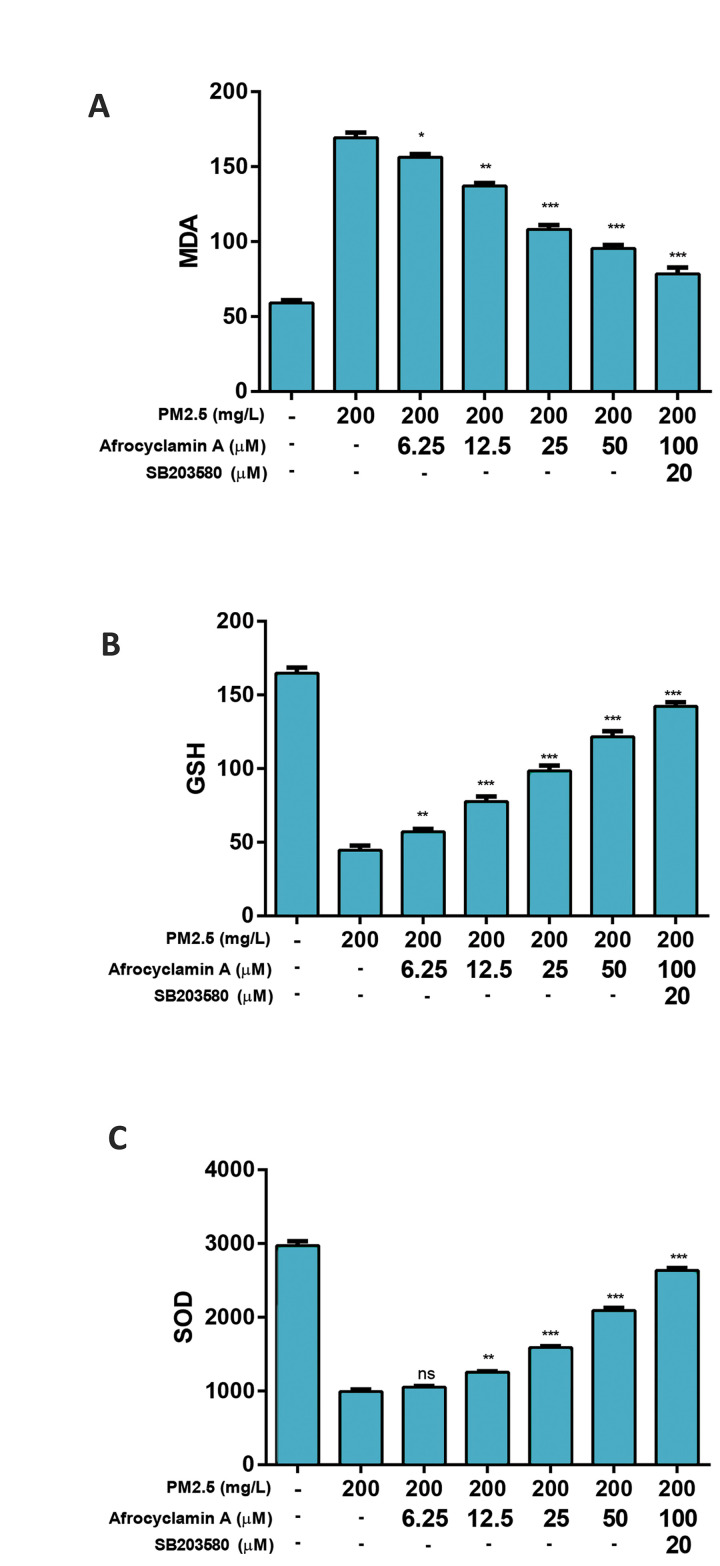
Effect of Afrocyclamin A on the antioxidant enzymes in VSMCs. **A.** Level of MDA, **B.** GSH, and C. SOD. The results are displayed as mean ± SEM (n=3). Compared to the PM2.5 (200 mg/l), *; P<0.05, **; P<0.01 and ***; P<0.01. VSMCs; Vascular smooth muscle cells, MDA; Malonaldehyde, GSH; Glutathione, SOD; Superoxide dismutase, and ns; Non significant.

Figure 2 shows the effect of Afrocyclamin A on the antioxidant parameters in VSMCs. PM2.5 (200 mg/l) increased the level of MDA as compared to control and treatment with Afrocyclamin A significantly (P<0.05) and dose-dependently reduced the level of MDA. 

An opposite trend was observed in SOD and GSH levels. PM2.5 (200 mg/l) reduced the level of SOD and GSH and treatment with Afrocyclamin A dose-dependently increased their level almost near to the control group. 

#### Effect of Afrocyclamin A on the level of endothelin-1, nitric oxide and vascular cell adhesion molecule-1 in vascular smooth muscle cells

**Fig.3:**
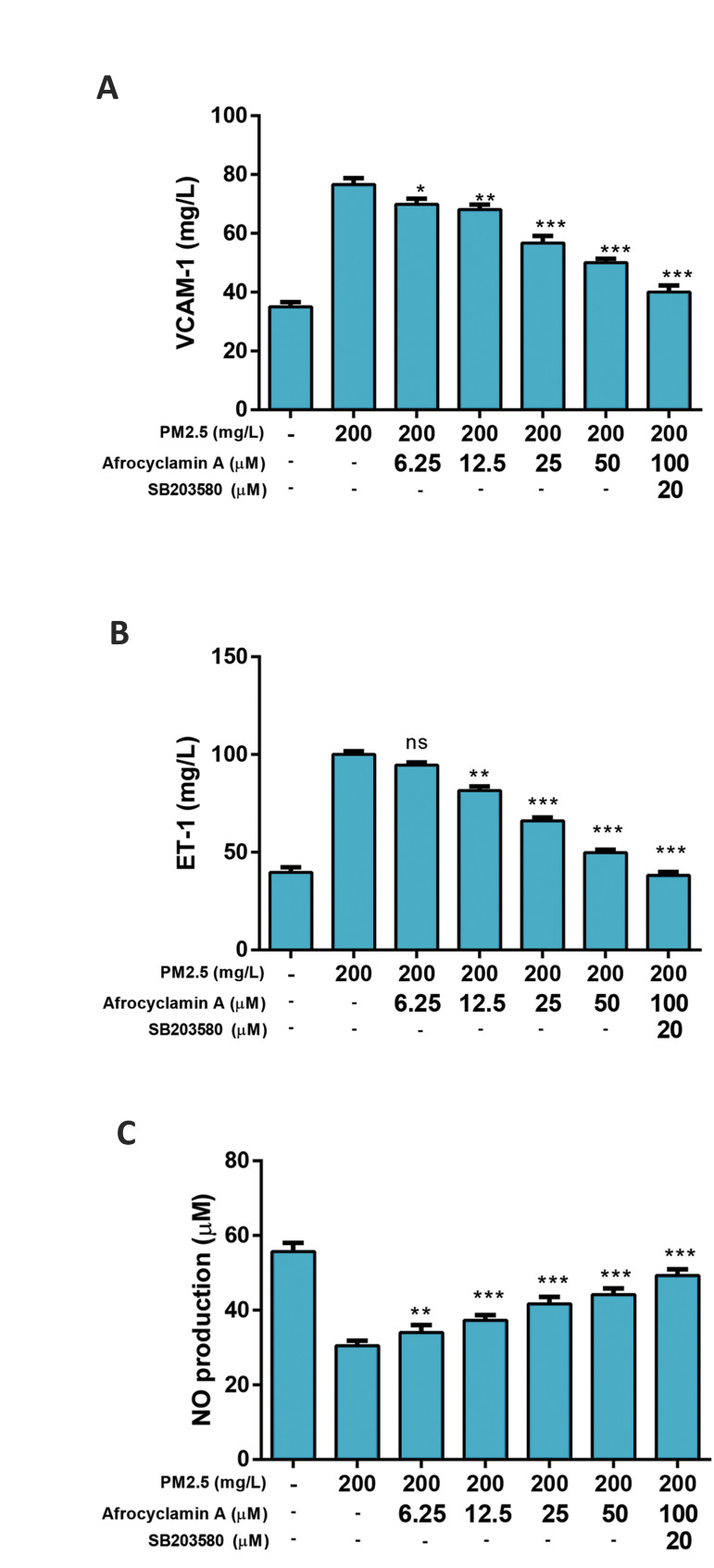
Effect of Afrocyclamin A on the antioxidant enzymes in VSMCs. **A.** Level of VCAM-1, **B.** ET-1 and C. NO. The results are displayed as mean ± SEM (n=3). Compared to the PM2.5 (200 mg/l), *; P<0.05, **; P<0.01 and ***; P<0.01. VSMC; Vascular smooth muscle cells, VCAM-1; Vascular cell adhesion molecule-1, ET-1; Endothelin-1, NO; Nitric oxide, and ns; Non significant.

Figure 3 exhibits the level of endothelin-1 (ET-1), NO and vascular cell adhesion molecule-1 (VCAM-1) in the treated and untreated cells. Compared to the untreated cells, PM2.5 considerably increased the level of ET-1, NO and VCAM-1 and treatment with Afrocyclamin A dose-dependently reduced the level of ET-1, NO and VCAM-1. These effects of Afrocyclamin A were increased by SB203580 as compared to the Afrocyclamin A-treated cell.

#### Effect of Afrocyclamin A on the cytokines in vascular smooth muscle cells

**Fig.4:**
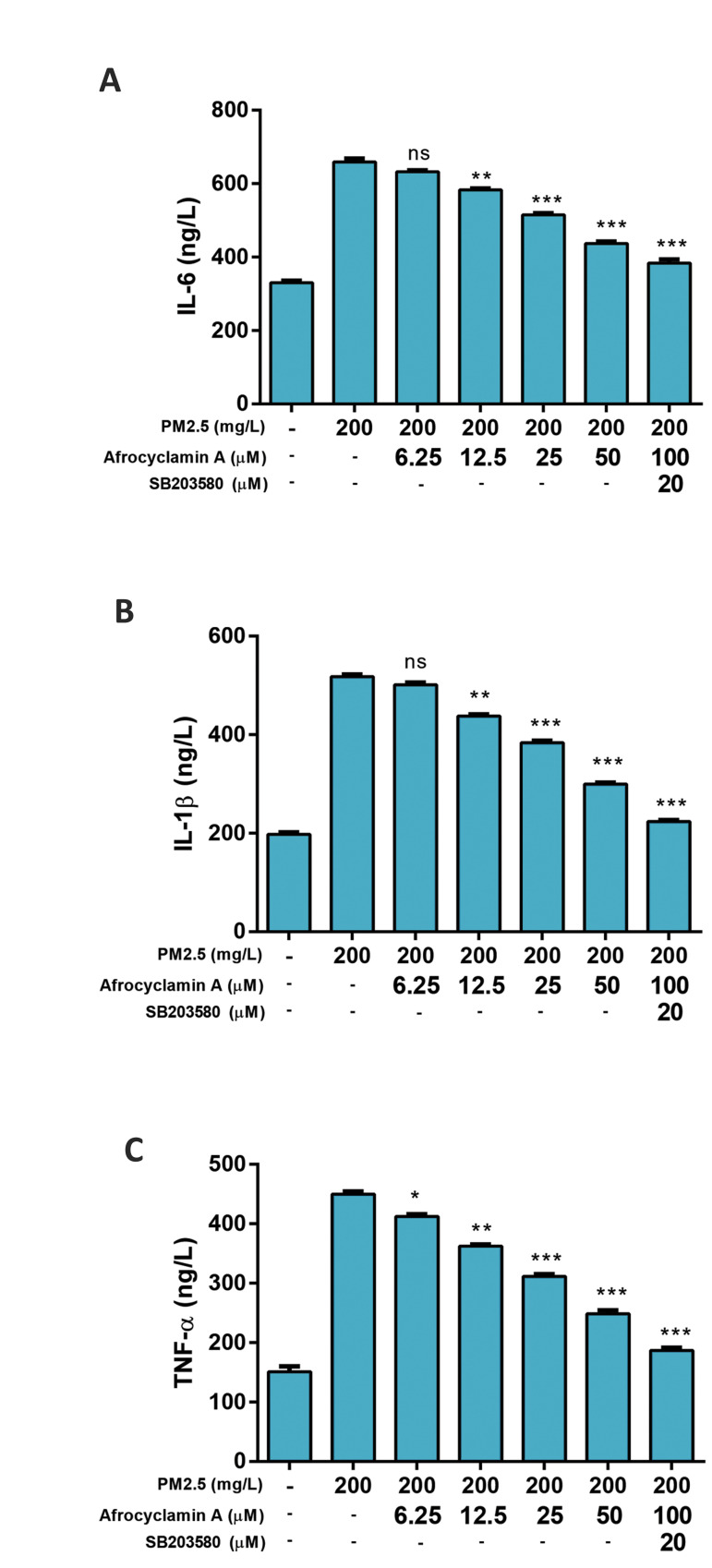
Effect of Afrocyclamin A on the pro-inflammatory cytokines in VSMCs. **A.** Level of IL-6, **B.** IL-1β, and **C.** TNF-α. The results are displayed as mean ± SEM (n=3). Compared to the PM2.5 (200 mg/l), *; P<0.05, **; P<0.01 and ***; P<0.01. VSMC; Vascular smooth muscle cells, IL-1β; Interleukin-1β, IL-6; Interleukin-6, TNF-α; Tumor necrosis factor-α, and ns; Non significant.

Compared to the untreated cells, the levels of pro-inflammatory cytokines such as TNF-α, IL-1β and IL-6, were increased in the PM2.5-treated cells. Afrocyclamin A considerably decreased the level of cytokines such as TNF-α, IL-1β and IL-6 in a dose-dependent manner. These effects of Afrocyclamin A were enhanced by SB203580 as compared to the Afrocyclamin A-treated cell (Fig .4). 

#### Effect of Afrocyclamin A on the expression of proliferating cell nuclear antigen and p-p38 MAPK in vascular smooth muscle cells

**Fig.5:**
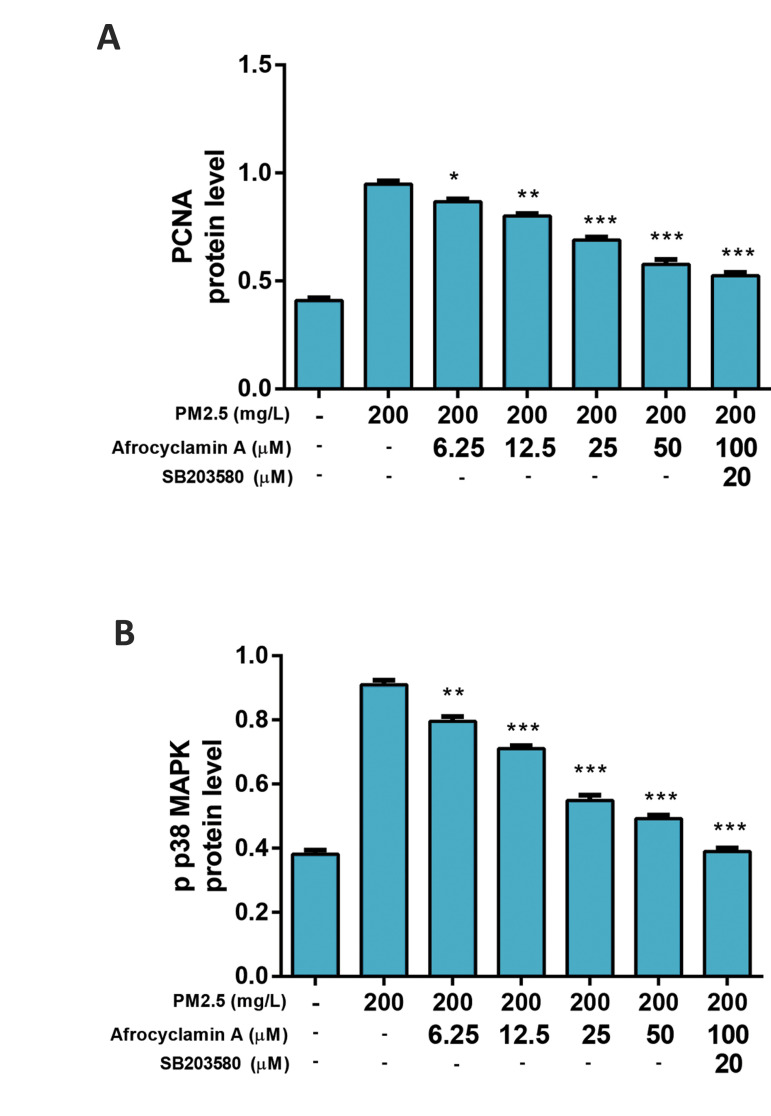
Showed the effect of Afrocyclamin A on the PCNA and p-p38MAPK in VSMCs. **A.** Showed the level of PCNA and **B.** p-p38 MAPK. Method described in the material and method section. The results are displayed as mean ± SEM (n=3). Compared to the PM2.5 (200 mg/L), *; P<0.05, **; P<0.01 and ***; P<0.01. VSMC; Vascular smooth muscle cells and PCNA; Proliferating cell nuclear antigen.

Figure 5 exhibits the effect of Afrocyclamin A on the expression of PCNA and p-p38 MAPK. Compared to the untreated cells, the PM2.5-treated cells demonstrated increased levels of PCNA and p-p-38 MAPK and Afrocyclamin A considerably decreased the level of PCNA and p-p38 MAPK in VSMCs. These effects of Afrocyclamin A were enhanced by SB203580 as compared to the Afrocyclamin A-treated cell.

## Discussion

Previous studies suggested that speedy industrialization and urbanization in China have led to a sharp boost in pollution emissions and energy consumption, especially in the metro city (10, 11). Due to urbanization, pollution is increased and coal consumption is increased during the winter season. According to the reports, only in Beijing, there is an increase in the concentration of PM2.5 particles due to continually increasing vehicle and coal use (12, 13). 

Previous reports showed that the particulate matter is a combination of different chemical compositions such as elemental nitrate, carbon, ammonium ion, silicon, sulfate, sodium ion and organic carbon matter (14, 15). According to the aerodynamic diameter, particulate matter is divided according to the size of particles as follows: <0.1 μm (PM 0.1), <2.5 μm (PM2.5), <10 μm and thoracic particles (>10 μm) (16). PM2.5 is very minute in size, which allows it to easily threat the human health by entering via trachea and going into the alveoli, penetrating via pulmonary air blood barrier, diffusing into the capillaries and finally entering the blood circulation (17). Consequently, the above discussed points suggest that PM2.5 can affect CVD and increase the CVD-related mortality. Due to increasing pollution, there is an increase in the incidence of CVD (18, 19). CVDs including atherosclerosis are related to the endothelial dysfunction, and alteration of CVDs risk factor leads to increased vascular function (20, 21). 

Previous researches suggested that the vascular calcification is a significant risk factor for cardiovascular mortality and morbidity and it is also predominant in the patients with atherosclerosis and diabetes (22, 23). Considering that the vascular calcification is related to the CVD risk factor, and various studies have attempted to interrupt the demonstration of disease. It was suggested that atherosclerosis is a progressive disease developed via deposition of fibrous plaque and lipids in the arteries (24, 25). The etiology of atherosclerosis is very complicated and its risk factors are hypertension, hyperlipidemia, smoking, lack of exercise and genetic defects (26). Various investigation suggested that PM2.5 may also take part in the expansion of CVD especially atherosclerosis (27, 28). It was shown that the endothelial dysfunction is considered a pathological condition, mostly produced by an imbalance between the vasoconstrictor and vasodilator substances, and this disproportion leads to damage of endothelium-dependent relaxation, which shows the functional characteristic of endothelial dysfunction (29, 30). All blood vessels play an important role in the switch of vascular tone partially via secretion of powerful vasodilators such as endothelium-derived hyperpolarizing factor (EDHF) and NO (31). The dysfunction of endothelial function is the 1st step towards the coronary arteriosclerosis disease, and long term exposure to PM2.5 was linked with the reduced level of NO-mediated endothelial function in a conduit artery independent of cardiovascular risk factors (31, 32). PM2.5 exposures resulted in increased level of inflammatory mediators and oxidative stress (33, 34). Currently, few studies suggest that plant-based drugs could decrease plasma calcification and arterial calcification concentration, but the underlying mechanism of action is still not clear. In the current experimental study, we scrutinized the antioxidant and anti-inflammatory effect of Afrocyclamin A against PM2.5-induced VSMCs.

During the expansion of atherosclerosis, the transformation of VSMCs from the inactive contractile phenotype towards the proliferative migratory phenotype into the plaque area to form a fibrous cap, is generally regarded as an important step in the formation of unstable atherosclerotic plaques (35). VSMCs drifted into the intima show an abnormally up-regulation in the production of extracellular matrix and proliferation, which further leads to the formation of the fibrous cap in atherosclerotic lesions. During atherosclerosis, the level of endothelial NO increased due to increase in the production of NO from the NO synthase (eNOS). Decreases in the level of NO, decrease the adhesion and aggregation of platelets and inflammatory cells. Decreased NO production plays a significant role in the expansion of leukocytes, which further increased the inflammation reaction and further increased the atherosclerotic plaque formation and instability (4, 35).

VCAM-1 (immunoglobulin-like glycoprotein), takes part in the adhesion of leukocytes to the endothelial cells and afterward, starts the transmigration into the arterial intima, and boosts the VSMCs proliferation via focal adhesion kinase pathway (36). It was shown that the pro-inflammatory cytokines such as IL-6, IL-1β and TNF-α, can induce VSMCs migration/proliferation and hypertrophic response, which can take part in the expansion of atherosclerosis (37). During atherosclerosis, the level of IL-6, IL-1β and TNF-α significantly increased and treatment with Afrocyclamin A dose-dependently reduced the level to almost near the control values. Oxidative stress plays a significant role in the development of atherosclerosis, and it is involved in the regulation of VSMCs migration/proliferation and differentiation (36). MDA (a marker of lipid peroxidation) acts as the endogenous lipid peroxidation and it is generated as the end product of lipid pre-oxidation (LPO). Other significant antioxidant enzymes such as SOD, play a role in the inhibition of neointima formation via attenuation of proliferation and migration of VSMCs. Other antioxidant enzymes like GSH, are reduced during atherosclerosis due to increased oxidative stress (38). Treatment with Afrocyclamin A significantly and dose-dependently altered the level of antioxidant enzymes.

It is well documented that endothelial cells, take part in the VSMCs hyperproliferation via MAPK-PI3K pathway (39). MAPKs are a group of signaling molecules that regulate apoptosis, proliferation, inflammatory reactions and differentiation via activating various downstream transcription factors. p38 MAPK is strongly activated in response to vascular damage, and signaling pathway of p38MAPK has been shown to affect VSMCs proliferation in response to proliferative factors via altering the progression of cell cycle linked proteins (40). According to their study, the effect of puerarin on the VSMCs proliferation is mediated via reduction of p38 MAPK signaling pathway. In our experimental study, Afrocyclamin A significantly inhibited the VSMCs proliferation via down-regulation of antioxidant and pro-inflammatory cytokines and down-regulated the p38 MAPK signaling pathway. 

## Conclusion

In this study, we observed that PM2.5 treatment significantly increased the VSMCs proliferation and increased the expression of p-p38MAPK, enhanced the level of IL-6, IL-1β, TNF-α, VCAM-1, MDA and reduced level of SOD, GSH and NO. The above-discussed results showed that PM2.5 might induce VSMCs proliferation through p38 MAPK signaling pathway activation. Afrocyclamin A significantly altered P38 MAPK and reduced the VSMCs proliferation. 
